# IFN-λ4 is associated with increased risk and earlier occurrence of several common infections in African children

**DOI:** 10.1038/s41435-021-00127-7

**Published:** 2021-04-13

**Authors:** Ludmila Prokunina-Olsson, Robert D. Morrison, Adeola Obajemu, Almahamoudou Mahamar, Sungduk Kim, Oumar Attaher, Oscar Florez-Vargas, Youssoufa Sidibe, Olusegun O. Onabajo, Amy A. Hutchinson, Michelle Manning, Jennifer Kwan, Nathan Brand, Alassane Dicko, Michal Fried, Paul S. Albert, Sam M. Mbulaiteye, Patrick E. Duffy

**Affiliations:** 1 Laboratory of Translational Genomics, Division of Cancer Epidemiology and Genetics, National Cancer Institute, National Institutes of Health, Bethesda, MD 20850 USA; 2 Laboratory of Malaria Immunology and Vaccinology, National Institute of Allergy and Infectious Diseases, National Institutes of Health, Bethesda, MD 20892 USA; 3 Malaria Research & Training Center, Faculty of Medicine, Pharmacy and Dentistry, University of Sciences Techniques and Technologies of Bamako, Bamako, Mali; 4 Biostatistics Branch, Division of Cancer Epidemiology and Genetics, National Cancer Institute, National Institutes of Health, Rockville, MD 20850 USA; 5 Cancer Genomics Research Laboratory, Frederick National Laboratory for Cancer Research, Frederick, MD 21702 USA; 6 Laboratory of Clinical Immunology and Microbiology, National Institute of Allergy and Infectious Diseases, NIH, Bethesda, MD 20892 USA; 7 Department of Surgery, University of California San Francisco, San Francisco, CA 94143 USA; 8 Infections and Immunoepidemiology Branch, Division of Cancer Epidemiology and Genetics, National Cancer Institute, National Institutes of Health, Bethesda, MD 20892-9776 USA

**Keywords:** Genetic variation, Infectious diseases

## Abstract

Genetic polymorphisms within the *IFNL3*/*IFNL4* genomic region, which encodes type III interferons, have been strongly associated with clearance of hepatitis C virus. We hypothesized that type III interferons might be important for the immune response to other pathogens as well. In a cohort of 914 Malian children, we genotyped functional variants *IFNL4*-rs368234815, *IFNL4*-rs117648444, and *IFNL3*-rs4803217 and analyzed episodes of malaria, gastrointestinal, and respiratory infections recorded at 30,626 clinic visits from birth up to 5 years of age. Compared to children with the rs368234815-TT/TT genotype (IFN-λ4-Null), rs368234815-dG allele was most strongly associated with an earlier time-to-first episode of gastrointestinal infections (*p* = 0.003). The risk of experiencing an infection episode during the follow-up was also significantly increased with rs368234815-dG allele, with OR = 1.53, 95%CI (1.13–2.07), *p* = 0.005 for gastrointestinal infections and OR = 1.30, 95%CI (1.02–1.65), *p* = 0.033 for malaria. All the associations for the moderately linked rs4803217 (*r*^2^ = 0.78 in this set) were weaker and lost significance after adjusting for rs368234815. We also analyzed all outcomes in relation to IFN-λ4*-*P70S groups. Our results implicate IFN-λ4 and not IFN-λ3 as the primary functional cause of genetic associations with increased overall risk and younger age at first clinical episodes but not with recurrence or intensity of several common pediatric infections.

## Introduction

Despite the tremendous progress, pediatric mortality remains unacceptably high. In 2015, of 5.9 million deaths before the age of five that occurred globally, 30% of deaths were caused by pneumonia, malaria, and diarrhea [1]. Thus, it is critical to identify and understand the factors affecting susceptibility and host response to common infections responsible for these deaths. The outcome of any infection is determined by a complex interplay between host and pathogen factors. One of the main host factors is the immune system, which can be affected by genetic polymorphisms in immune response genes.

For example, variable ability to clear hepatitis C virus (HCV) infection is associated with polymorphisms in the genetic locus on chromosome 19 that encodes the family of type III interferons (IFNs) [2–6]. These IFNs provide a localized immune response at the sites of pathogen entry, such as at the epithelial surfaces of the respiratory and gastrointestinal tracts, as well as in the liver [7, 8]. If this first line of defense provided by type III IFNs is successful, it decreases the necessity of a much stronger and systemic immune response provided by type I IFNs (IFNα and IFNβ) and type II IFN (IFNɣ).

Humans have four type III IFNs, all encoded within the 70 kb region on chromosome 19. Three of these IFNs (IFN-λ1-3) can be inducibly generated in all the individuals, while IFN-λ4 is produced only in about 50% of the world population, including 90% of individuals of African ancestry, versus only 50% of Europeans and 10% of Asians [6, 9]. The ability to produce IFN-λ4 is an ancestral trait, which appears to be under negative selection in humans [10]. The reasons for this selection are unclear but could be related to poor clearance of some deadly infections in the past. Thus, historic infections might have shaped the genetic landscape of this locus. On the other hand, the variable production of IFN-λ4 might have influenced and still be influencing susceptibility to infections and affecting clinical outcomes.

The production of IFN-λ4 is controlled by a frameshift dinucleotide polymorphism, rs368234815-dG, within the first exon of the *IFNL4* gene. The open reading frame for IFN-λ4 protein is created by the dG allele, whereas the TT allele results in the truncated, non-functional protein (IFN-λ4-Null) [6]. Once IFN-λ4 is produced in the presence of the rs368234815-dG allele, it may carry a missense variant rs117648444-A/G (P70S) within exon 2; this protein change was shown to attenuate the antiviral activity of IFN-λ4 [11]. The combination of the two *IFNL4* SNPs (rs368234815 and rs117648444) stratifies all individuals in groups of those not producing any IFN-λ4 protein, producing only weak IFN-λ4-S70 or strong IFNλ4-P70 protein. The ability to produce strong IFNλ4-P70 protein was associated with impaired HCV clearance [11] and reduced survival after allogeneic hematopoietic stem cell transplantation [12], indicating the negative functional impact of the biologically active IFN-λ4.

Although rs368234815-dG is considered the causal variant due to its direct functional effect on the production of IFN-λ4 protein, whose biological activity might be modified by rs117648444 (P70S), additional polymorphisms in the *IFNL3/IFNL4* region may also demonstrate significant associations because of population-dependent moderate to high linkage disequilibrium in this genomic region. One of these linked variants is a single nucleotide polymorphism (SNP) rs4803217, which is located within the 3′UTR of *IFNL3*; this variant was proposed to be functional and affecting IFN-λ3 protein by modulating the stability of *IFNL3* mRNA [13]. Another polymorphism, rs12979860, also known as the *IL28B* (*IFNL3*) marker, is located within the first intron of *IFNL4* and often used as a proxy for the functional rs368234815 but without any known functional effect.

The strong associations between the *IFNL3*/*IFNL4* genetic variants and HCV clearance have been well established and replicated by multiple studies [2–6]. Beyond HCV, the *IFNL4* genetic polymorphisms (rs12979860 and rs368234815) have been associated with the clearance of respiratory RNA viruses in children in Rwanda [14]. To further explore the hypothesis that type III IFNs might impact the immune response to diverse pathogens, we analyzed detailed health records of 914 Malian children and genotyped their DNA for the functional polymorphisms *IFNL4*-rs368234815, *IFNL4-*rs117648444, and *IFNL3*-rs4803217. We analyzed clinical data collected during 30,626 routine and walk-in clinic visits from birth to up to 5 years of age. Specifically, we analyzed episodes of infections classified as malaria (overall, severe and non-severe), gastrointestinal, and respiratory infections and evaluated their occurrence during follow-up time (ever/never), time-to-first episode, and the count of independent episodes. Our results provide new clues on the role of type III IFNs, and IFN-λ4, specifically, in genetic control of the immune response to several common infections in African children.

## Methods

### The Mali longitudinal birth cohort

The study used data and samples collected by the longitudinal birth cohort of 914 children enrolled between 2010 and 2016 in Ouelessebougou Community Health Center (CESCOM), located 80 km south of Bamako, Mali. At their antenatal visits, pregnant women aged 15–45 years were invited to participate in the study. The eligibility criteria included an absence of a chronic, debilitating illness and residence in Ouelessebougou district for at least 12 months before enrollment. The newborn children of the participating mothers were enrolled at delivery, and cord blood was collected. The study site is located in an area of intense but highly seasonal malaria transmission. The follow-up protocol included visits to the clinic scheduled monthly during the high malaria transmission season (June–December) and every two months during the dry season (January–May), as well as ad hoc walk-in clinic visits for any acute symptoms that could not wait for the next regularly scheduled follow-up visit. Clinical information was collected during all visits by project clinicians and recorded using standardized forms.

Since the primary goal of this birth cohort was to explore host and parasite factors that influence susceptibility to malaria infection and disease during pregnancy and early childhood, detailed malaria-related symptoms were recorded at each visit. Per study protocol, malaria infection was defined as positivity for *P. falciparum* malaria parasite detected by thick blood smear microscopy, with or without clinical symptoms. Clinical malaria was defined as fever >37.5 °C, presence of malaria-related symptoms, and either a positive Rapid Diagnostic Test for malaria antigens or visualized malaria parasites on thick blood smear microscopy. Severe malaria was defined as *P. falciparum* parasitemia and at least one of the following World Health Organization criteria for severe malaria—2 or more convulsions in the past 24 h, prostration (inability to sit unaided or in younger infants inability to move/feed), hemoglobin <5 g/dl, respiratory distress (hyperventilation with deep breathing, intercostal recessions and/or irregular breathing), or coma (Blantyre score <3). The presence of severe malaria symptoms but with negative tests for malaria antigens or parasites was attributed to other diseases. All other parasitemia episodes, including those that were asymptomatic, were classified as non-severe malaria. For ever/never analyses, the non-severe malaria group included children with malaria history who never had a severe malaria episode, while the severe malaria group included children with at least one severe malaria episode, regardless of the number of non-severe malaria episodes.

Standardized clinical forms also captured other symptoms, such as diarrhea and cough, and other signs of respiratory distress (sputum production, dyspnea, tachypnea, abnormal auscultatory findings). Using a syndromic approach, we attributed diarrhea to gastrointestinal infections and cough/respiratory distress to respiratory infections. Although both gastrointestinal and respiratory infections could be caused by bacterial, viral, parasitic, or fungal infections, this information was not collected by the study, and a more detailed etiology could not be explored. All children received treatment based on their symptoms. Episodes of the same infection were considered independent if they occurred more than 28 days apart.

Before enrollment, written informed consent was obtained from all study participants after receiving written and oral study explanations from a study clinician in their native language; the parents/guardians provided consent on behalf of their children. All methods were carried out in accordance with relevant guidelines and regulations. The study procedures were ethically approved by the Institutional Review Board of the National Institute of Allergy and Infectious Diseases (NIAID) at the US National Institutes of Health (ClinicalTrials.gov ID NCT01168271), and the Ethics Committee of the Faculty of Medicine, Pharmacy and Dentistry at the University of Bamako, Mali.

### DNA extraction and genotyping

Genomic DNA was extracted from cord blood clots using the QIAsymphony automation system with protocol Blood_200_V7_DSP and the DSP DNA Mini Kit (Qiagen) by the Cancer Genome Research Laboratory of the DCEG/NCI. The DNA samples were tested by microsatellite fingerprinting (AmpFLSTR Identifiler, ThermoFisher) to confirm gender concordance with study records and exclude any mixed-up or contaminated samples.

DNA samples were genotyped for *IFNL4*-rs368234815, *IFNL4-*rs117648444, and *IFNL3*-rs4803217 by custom TaqMan genotyping assays as previously described [6, 15] (Supplementary Fig. S1). *HBB* polymorphisms rs334-T/A, *HBB*-Glu7Val (HbS, encoding hemoglobin variants A/A, A/S, and S/S) and rs33930165, *HBB*-Glu7Lys (HbC, encoding hemoglobin variants A/A, A/C, and C/C) were genotyped by Sanger sequencing as previously described [16] (Supplementary Fig. S2). Approximately 10% of all samples were genotyped in technical duplicates, which were 100% concordant for all markers. The distributions of *IFNL3* and *IFNL4* SNPs did not deviate from Hardy–Weinberg equilibrium (HWE). All genotyping and sequencing were done in the Laboratory of Translational Genomics (LTG, DCEG, NCI) blinded to demographic and clinical data. Merging with phenotypes and outcomes was done externally (NIAID) after all the genotyping was complete. Of the initial cord blood clots from 993 children, 21 (2.1%) were excluded due to poor quantity/quality of the extracted DNA. Of the remaining 972 samples, 38 samples (3.9%) were excluded due to discordant *HBB* status between the study records (determined based on gel electrophoresis) and DNA genotyping, 18 samples (1.9%) were excluded due to sex discordance between study records and Identifiler profiles and two samples (0.2%)—due to failed rs368234815 genotyping.

### Statistical analysis

For each infection outcome, we considered the occurrence of at least one episode during the follow-up time (ever/never), time-to-first episode, and the count of independent episodes. The main outcome predictors were genetic variants *IFNL4*-rs368234815 and *IFNL3*-rs4803217, which were evaluated assuming the additive and genotypic genetic models, using the TT/TT genotype or TT allele (IFN-λ4-Null) for rs368234815 and G/G genotype or G allele for rs4803217 as reference groups. Haplotypes and linkage disequilibrium metrics (*D*′ and *r*^2^) between the markers were analyzed and plotted using Haploview 4.2 [17].

The occurrence of infection episodes (never/ever) was analyzed for both polymorphisms separately and in the same model using logistic regression models and adjusting for the follow-up time (weeks). Based on rs368234815 and rs117648444, all children were stratified into four groups: those not producing any IFN-λ4 protein (Null), producing only weak IFN-λ4-S70 protein, and one or two copies of the strong IFN-λ4-P70 protein. *HBB* status coded as 0, 1, and 2 for AA (wild-type) vs. sickle-cell trait (A/S or A/C) vs. sickle-cell disease (S/S, S/C or C/C) was used as a covariate for all malaria-related analyses; sex and birth month (as a proxy for seasonal effects) were tested but not significantly associated with any outcomes. Logistic regression models were used to evaluate the associations of *IFNL4*-rs368234815 with infections of interest, adjusting for the occurrence of other infections (ever/never) to examine whether these associations were specific to particular infection types.

Time-to-first episode of each infection was analyzed with Kaplan-Meyer plots and Cox proportional hazards regression models. The number of infection episodes during follow-up was analyzed using Poisson models with overdispersion (Quasi-likelihood), adjusting for the length of follow-up as an offset in the model (offset = log(LastVisitAge)).

Since the study did not have permission to perform genome-wide analyses of genetic variants that could be used to adjust for possible population stratification, we used self-reported paternal tribe information as a proxy. Of the 21 tribes, four tribes accounted for 92.5% of all the children: Bamana—72.3%, Peulh/Fulani—9.2%, Soninke—6.0%, and Malinke—5.0%. The reported paternal tribe (for all 21 tribes) was used as a random effect variable for logistic regression analyses (glmer package), Cox regression analyses with gamma frailty, and the analyses of infection episode counts per year (glmmPQL package). All the analyses were performed using specified packages in R (https://cran.r-project.org/). The results were not adjusted for multiple testing.

## Results

### The cohort

The Mali birth cohort included 914 children with available genotype, demographic and clinical data (Table 1). Based on data collected by this longitudinal study, we focused on three main outcomes: malaria (overall infection, severe, and non-severe), gastrointestinal infections, and acute respiratory infections. Malaria diagnoses were specific and required plasmodium detection, with or without other symptoms. Gastrointestinal and respiratory infections were defined based on the syndromic approach, using diarrhea and coughing/respiratory distress as respective proxies.

**Table 1:** Demographic characteristics of 914 children enrolled in the Mali birth cohort.

Sex	
Males, *n*, %	467 (51.1)
Females, *n*, %	447 (48.9)
Follow up characteristics	
Median time, weeks (range)	140 (52–264)
Median number of visits per child (range)	31 (5–81)
Children with at least one episode of infection during follow up, *n* (%) of all	
Gastrointestinal infection (GII)	805 (88.1)
Respiratory infection (RI)	893 (97.7)
Malaria overall	696 (76.1)
Severe malaria (SM)	102 (11.2)
Non-severe malaria (NSM)	686 (75.1)

Episodes of any infection (malaria, gastrointestinal or respiratory) were reported at 12.7% of the routine and 76.3% of the walk-in visits (Table 2). Thus, most infection episodes were acute and sufficiently severe to require an emergency visit to the clinic without waiting for the next regular scheduled check-up visit. The overall frequency of malaria infection at any visit was 10.6% (5.4% at routine and 19.9% at walk-in visits); gastrointestinal infection was reported at 8.4% of all visits (1.8% at routine and 20.2% at walk-in visits), and respiratory infection was reported at 21.0% of all visits (6.5% at routine and 47.4% at walk-in visits) (Table 2). No infection was reported at most visits (*n* = 19,808, 64.7%), while one and two infection types were reported at 9407 (30.7%) and 1380 (4.5%) visits, respectively. Only 31 visits (0.1%) reported all three infection types at the same visit.

**Table 2:** Distribution of clinic visits for 914 children from the Mali birth cohort study.

	Clinic visits, *n*			
	All visits#	Routine	Walk-in	Other
	30,626	18,654	10,884	1088
Visits reporting any infection, *n*,	10,818	2377	8303	138
% of all visits	35.3	12.7	76.3	12.7
Visits with malaria, *n*,	3,260	1,015	2,167	78
% of malaria visits	100.0	31.1	66.5	2.4
% of all infections	10.6	5.4	19.9	7.2
Visits with GII, *n*	2,563	342	2204	17
% of GII visits	100.0	13.3	86.0	0.7
% of all infections	8.4	1.8	20.2	1.6
Visits with RI, *n*	6,437	1,221	5,161	55
% of RI visits	100.0	19.0	80.2	0.9
% of all infections	21.0	6.5	47.4	5.1

Malaria—positivity by blood smear test with/without additional clinical symptoms, includes both severe (SM) and non-severe malaria (NSM). Other visits include post-disease wellness check-ups.

*GII* gastrointestinal infections, *RI* respiratory infections.

These infections were often detected in combination: respiratory infection and malaria was the most common co-infection combination (5.3% of routine and 8.2% of walk-in visits), while malaria and gastrointestinal infection was the least common combination, with 0.8% of routine and 1.6% of walk-in visits (Supplementary Table S1). Logistic regression analyses showed that infections tended to be non-redundant, and any infection significantly decreased the risk of having any other infection reported at the same visit (Supplementary Table S2).

### Distribution of the HBB, IFNL4, and IFNL3 polymorphisms in the Malian children and the populations of the 1000 Genomes Project

In the total set of 914 Malian children, the *IFNL4*-rs368234815 alleles (dG-72.2% and TT-28.8%), and *IFNL3*-rs4803217 alleles (T-66.7% and G-33.3%) were in moderate linkage disequilibrium (*D*’ = 0.98 and *r*^2^ = 0.78), *IFNL4*-rs117648444 alleles were present at A-10% and G-90% frequencies (Supplementary Table S3); the A allele was found only in one haplotype, with rs368234815-dG and rs4803217-T allele (Supplementary Fig. S3).

The wild-type *HbB*-A/A genotype was present in 80.5% of children; the sickle cell genotypes (SCT, HbS-A/S, or HbC-A/C) in 18.6%, and homozygous variant genotypes associated with sickle cell disease – (SCD, S/S, S/C or C/C) in 0.9% of the children.

The allele and genotype frequencies of *HBB, IFNL3* and *IFNL4* polymorphisms were comparable to those in the populations of African ancestry in the 1000 Genomes Project, especially to the populations from West-Africa (Supplementary Table S3). The distribution of alleles/genotypes of these variants in the four main tribes in Mali is also presented (Supplementary Table S3).

### Associations between IFNL4 and IFNL3 polymorphisms and infection episodes in the Malian children

We analyzed the risk of an infection episode reported at any clinic visit from birth through up to 5 years of follow-up (ever/never analysis) and present the results adjusted for the tribes; unadjusted results are also presented in corresponding tables/figures.

The rs368234815-dG allele was associated with an increased risk of gastrointestinal infections (with a per-allele OR = 1.53, 95%CI (1.13–2.07), *p* = 0.005), malaria (OR = 1.30, 95%CI (1.02–1.65), *p* = 0.033), non-severe malaria (OR = 1.28, 95%CI (1.00–1.63), *p* = 0.047), but not of respiratory infections (OR = 1.20, 95%CI (0.63–2.29, *p* = 0.58) (Table 3). Gastrointestinal and respiratory symptoms might overlap with clinical manifestations of malaria. However, the associations of *IFNL4*-rs368234815 with these infections appeared independent, as mutual conditioning on these infections did not significantly alter the results (Supplementary Table S4).

**Table 3:** Associations between IFNL4-rs368234815 and infections in 914 children from the Mali birth cohort study.

Infection			rs368234815 genotypes			rs368234815 alleles		Ever/never^a^		Ever/never adj^b^	
	Total % of total	Total	TT/TT	TT/dG	dG/dG	TT	dG	OR	*P* value	OR	*P* value
		*n*, %	*n*, %	*n*, %	*n*, %	*n*, %	*n*, %				
		914	77	373	464	527	1301				
			8.4	40.8	50.8	28.8	71.2				
Malaria^c^	Never	218	24	99	95	147	289	Ref		Ref	
	23.9		11.0	45.4	43.6	33.7	66.3				
	Any	696	53	274	369	380	1012	**1.32 (1.04–1.68)**	**0.022**	**1.30 (1.02–1.65)**	**0.033**
	76.1		7.6	39.4	53.0	27.3	72.7				
	SM	102	4	44	54	52	152	**1.53 (1.01–2.30)**	**0.044**	1.48 (0.98–2.24)	0.061
	11.2		3.9	43.1	52.9	25.5	74.5				
	NSM	594	49	230	315	328	860	**1.30 (1.02–1.65)**	**0.035**	**1.28 (1.00–1.63)**	**0.047**
	65.0		8.3	38.7	53.0	27.6	72.4				
GII	Never	109	16	49	44	81	137	Ref		Ref	
	11.9		14.7	45.0	40.4	37.2	62.8				
	Ever	805	61	324	420	446	1164	**1.50 (1.11–2.03)**	**0.0079**	**1.53 (1.13–2.07)**	**0.0054**
	88.1		7.6	40.3	52.2	27.7	72.3				
RI	Never	21	4	7	10	15	27	Ref		Ref	
	2.3		19.1	33.3	47.6	35.7	64.3				
	Ever	893	73	366	454	512	1274	1.20 (0.63–2.29)	0.58	1.20 (0.63–2.28)	0.58
	97.7		8.2	41.0	50.8	28.7	71.3				

*GII* gastrointestinal infections, *RI* respiratory infections, *any malaria* positivity by blood smear test with/without additional clinical symptoms, *SM* severe malaria, *NSM* non-severe malaria, in bold - significant associations.

^a^Logistic regression analysis based on additive genetic models and adjusting for the duration of follow-up (weeks).

^b^Additionally adjusting for tribes.

^c^Results for malaria are also adjusted for HBB status.

**Fig. 1:**
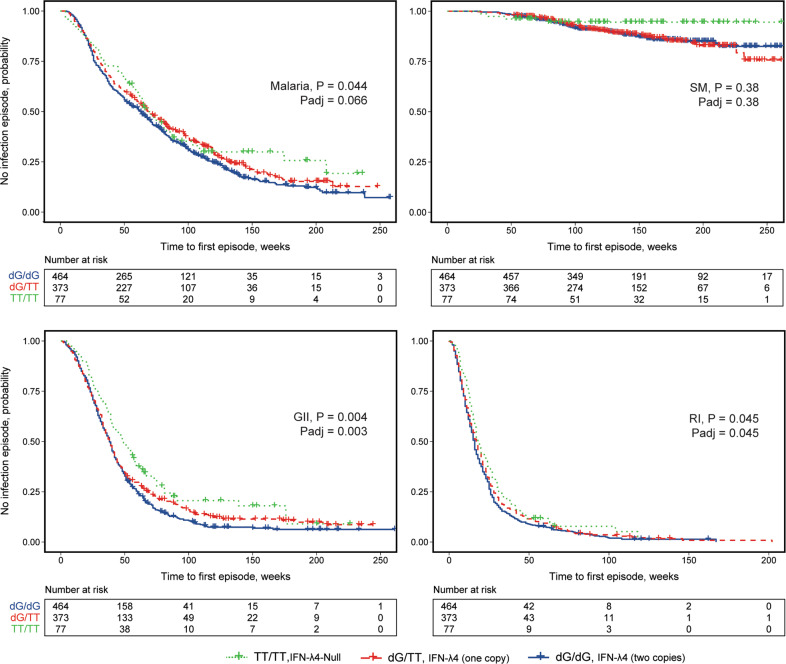
Time-to-first episode of infections in relation to *IFNL4*- rs368234815 polymorphism in 914 children from the Mali birth cohort study. Malaria – positivity by blood smear test with/without additional clinical symptoms, includes both severe (SM) and non-severe malaria; GII—gastrointestinal infections; RI—respiratory infections. The plots are for Kaplan–Meyer analysis, *P* values are for Cox proportional hazards regression models with per-allele linear trends, for malaria—adjusting for *HBB* status. *P*adj—additionally adjusting for sex and paternal tribes.

We also analyzed the association between *IFNL4*-rs368234815 and time-to-first episode of each infection, which represents the earliest occurrence of symptoms of sufficient severity to require a visit to the clinic. The most significant association was observed for the *IFNL4*-rs368234815 and time-to-first episode of gastrointestinal infections (per-allele *p* = 0.003) (Fig. 1). The association was marginally significant for respiratory infections (*p* = 0.045), but not for malaria overall and severe malaria (Fig. 1). The duration of follow-up did not differ significantly for children with different *IFNL4* genotypes (Supplementary Table S5).

The number of episodes reported for each infection during follow-up was the lowest for children with the IFN-λ4-Null genotype, but this was significant only in the dominant genetic model for gastrointestinal infections (*p* = 0.03, Supplementary Table S6). All the associations for *IFNL3*-rs4803217 were weaker than for rs368234815 (Supplementary Table S7, Supplementary Fig. S4) and lost significance in the models that included both variants, while most of the associations for rs368234815 remained significant (Table 4).

**Table 4:** Associations between IFNL4-rs368234815 and IFNL3-rs4803217 with infections in 914 children from the Mali birth cohort study.

Infection		*IFNL4*-rs368234815^a^		*IFNL3*-rs4803217^a^		Models with both variants^c^			
						rs368234815		rs4803217	
		OR, 95%CI	*P* val	OR, 95%CI	*P* val	OR, 95%CI	*P* val	OR, 95%CI	*P* val
Malaria^b^	Never	Ref		Ref		Ref		Ref	
	Any	**1.30**	**0.033**	1.19 (0.94–1.50)	0.15	**1.73 (0.96–3.10)**	**0.068**	0.74 (0.42–1.31)	0.30
		**(1.02–1.65)**							
	SM	**1.48**	**0.061**	**1.52 (1.01–2.27)**	**0.044**	1.04 (0.37**–**2.88)	0.94	1.46 (0.54**–**3.98)	0.45
		**(0.98–2.24)**							
	NSM	**1.28**	**0.047**	1.15 (0.91**–**1.46)	0.25	**1.87 (1.03–3.38)**	**0.039**	0.67 (0.37**–**1.19)	0.17
		**(1.00–1.63)**							
GII	Never	Ref							
	Ever	**1.53**	**0.005**	**1.35 (1.00–1.82)**	**0.047**	**2.34 (1.06–5.19)**	**0.036**	0.63 (0.29**–**1.39)	0.26
		**(1.13–2.07)**							
RI	Never	Ref							
	Ever	1.2 (0.63**–**2.28)	0.58	1.24 (0.66**–**2.33)	0.51	0.94 (0.23**–**3.90)	0.93	1.31 (0.32**–**5.31)	0.71

*any malaria* positivity by blood smear test with/without additional clinical symptoms; *SM* severe malaria; *NSM* non-severe malaria. $-additionally adjusting for tribes. In bold - significant associations.

^a^Logistic regression analysis based on additive genetic models and adjusting for the duration of follow-up (weeks).

^b^Results for malaria are adjusted for HBB status.

^c^Results for models including both variants.

## Modifying effect of IFN-λ4-P70S groups on associations with infections

Previous studies in Europeans classified all individuals into three groups based on two SNPs (rs368234815 and rs117648444) as those not producing any IFN-λ4 protein (Null), producing only weak IFN-λ4-S70 protein or producing at least one copy of the strong IFN-λ4-P70 protein [11, 12]. Because the missense variant rs117648444-A (P70S) within exon 2 of *IFNL4* exists only as a part of haplotype with rs368234815-dG allele (Supplementary Fig. S3), rs117648444 should only be analyzed together with rs368234815 (reviewed in [9]). In individuals of African ancestry, rs368234815-dG is a major allele (in contrast with Europeans and Asians), allowing further separation of the last group into individuals producing one versus two copies of the strong IFN-λ4-P70 protein; thus, we stratified all children in four groups instead of classification into three groups used in Europeans [11, 12].

**Fig. 2:**
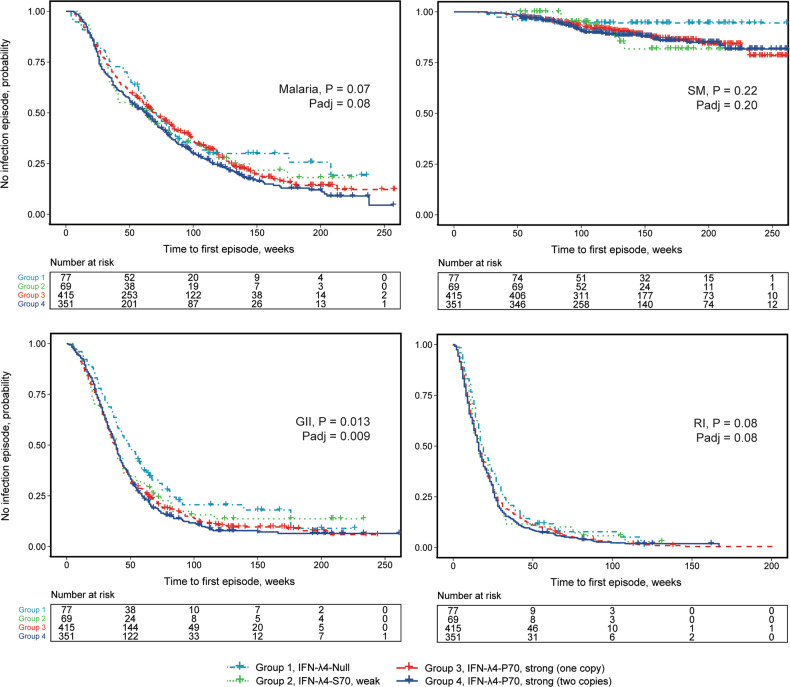
Time-to-first episode of infections in relation to IFN-λ4-P70S group status in 914 children from the Mali birth cohort study. Malaria – positivity by blood smear test with/without additional clinical symptoms, includes both severe (SM) and non-severe malaria; GII – gastrointestinal infections; RI – respiratory infections. The plots are for Kaplan-Meyer analysis, *P* values are for Cox proportional hazards regression models with per-group linear trends, for malaria – adjusting for *HBB* status. Padj – additionally adjusting for sex and paternal tribes.

Table 5 shows that compared to the IFN-λ4-Null group, the risks of gastrointestinal infection and malaria but not respiratory infection were increased in the following order: weak IFN-λ4-S70 < one copy of strong IFN-λ4-P70 < two copies of strong IFN-λ4-P70 protein. Similarly, IFN-λ4-P70S group classification was significantly associated with an earlier occurrence of the first GII episode (*p* = 0.009, Table 5 and Fig. 2). The numbers of infection episodes were not significantly associated with IFN-λ4-P70S groups (Supplementary Table S8).

**Table 5:** Association of IFN-λ4-P70S groups with infections in Mali birth cohort of 914 children.

					GII		RI		Malaria overall		Malaria severe		Malaria non-severe	
Group	rs368234815	rs117648444	IFN-λ4 status	*N* (%) All, *n* = 912 (100%)	OR (95% CI)	*P* value	OR (95% CI)	*P* value	OR (95% CI)	*P* value	OR (95% CI)	*P* value	OR (95% CI)	*P* value
1	TT/TT	G/G	IFN-λ4-Null	77 (8.4)	ref	ref	ref	ref	ref	ref	ref	ref	ref	ref
2	TT/dG	A/G	Weak IFN-λ4-S70	69 (7.6)	1.36	0.49	0.87	0.86	1.14	0.73	2.27	0.25	1.08	0.84
	dG/dG	A/A			(0.57**–**3.22)		(0.17**–**4.35)		(0.54**–**2.40)		(0.55**–**9.31)		(0.51**–**2.30)	
3	TT/dG	G/G	One copy of strong	415 (45.5)	1.93	0.045	3.29	0.09	1.23	0.46	2.54	0.12	1.16	0.61
	dG/dG	A/G	IFN-λ4-P70		(1.01**–**3.67)		(0.82**–**13.21)		(0.71**–**2.15)		(0.79**–**8.17)		(0.66**–**2.03)	
4	dG/dG	G/G	Two copies of strong IFN-λ4-P70	351 (38.5)	2.35	0.012	1.57	0.48	1.69	0.07	3.51	0.037	1.58	0.11
					(1.21**–**4.59)		(0.44**–**5.55)		(0.95**–**3.00)		(1.07**–**11.5)		(0.89**–**2.82)	
Trend, per group					**1.33**	**0.008**	1.21	0.38	**1.2**	**0.036**	**1.41**	**0.030**	1.18	0.062
					**(1.08–1.64)**		(0.78**–**1.88)		**(1.01–1.43)**		**(1.03–1.93)**		(0.99**–**1.41)	
Time-to-first episode of infection														
Log-rank (Mantel–Cox)						**0.009**		0.083		0.078		0.20		0.13

In bold—significant associations.

## Discussion

We leveraged extensive follow-up data from a large and well-characterized birth cohort from Mali with genetic analysis of three polymorphisms functionally relevant for two type III IFNs, IFN-λ3 and IFN-λ4. We showed that, compared to IFN-λ4-Null children, children with genetic ability to produce IFN-λ4, and especially those producing functionally strong IFN-λ4-P70 protein, were more likely to experience gastrointestinal infections, manifested by diarrhea, with the first infection episode occurring at a younger age. Although weaker, we also observed a similar trend for malaria and respiratory infections.

While using diarrhea and cough/respiratory distress as proxies for gastrointestinal and respiratory infections limits the etiological inferences on the observed genetic associations, these patterns are informative when considering the most common causes of these manifestations. For example, in young children, acute diarrhea with or without vomiting is likely to be of viral etiology, caused by infection with rotavirus (RoV), norovirus (NoV) or astrovirus (AstV) [18, 19], or their combinations [20]. In Rwanda, rotavirus was detected in rectal swabs of 36.9% of children under five seeking clinical help for acute gastroenteritis [21]. In 2016 alone, RoV was estimated to have caused >128,500 deaths of children under five [22]. Of all cases of acute gastroenteritis, NoV and AstV are estimated to account for 20% and 4–10%, respectively [19, 23]. Although these infections are ubiquitous and in healthy individuals spontaneously clear within days, viral clearance may take longer in younger children, especially in those affected by malnutrition and other infections. Extended severe dehydration due to diarrhea can be fatal, especially in infants.

Because human enteric viruses are difficult to study in vitro [24, 25], most conclusions about the immune response to these infections are drawn based on studies of murine viruses using mouse models. However, in contrast with humans who can produce either three or four type III IFNs (IFN-λ1, 2, 3 in all and IFN-λ4 in a subset of individuals), the mouse genome invariably encodes only IFN-λ2 and IFN-λ3 (collectively referred to as IFN-λ). Despite differences in the repertoire of human and mouse type III IFNs and notable variation between viral strains and mouse models [26], intact type III IFNs signaling was identified as critical for controlling enteric viral infections [26–31].

The higher risk and earlier episodes of gastrointestinal infections in children with *IFNL4*-rs368234815-dG allele might indicate an impaired immune response in these children due to the production of IFN-λ4. However, if diarrhea can be considered as a mechanism to purge those pathogens from the gastrointestinal tract, these results might also indicate an enhanced innate immune response, as has been suggested based on mouse studies [22]. The magnitude of this IFN-λ4-mediated immune response might be reflected in diarrhea severity or frequency, but our study did not have enough information to test this hypothesis.

Similarly, we broadly defined respiratory infections using cough/respiratory distress manifestations regardless of their etiology. Respiratory infections cause an estimated 1.9 million deaths annually in children under five, with 70% of these deaths occurring in Africa and Southeast Asia [32]. Some of these deaths are caused by infections with the respiratory syncytial virus (RSV) and influenza, also linked with the immune response mediated by type III in the human and murine nasal epithelium [33–36]. These studies suggested that type III IFNs represent the first line of defense from respiratory RNA viruses, and treatment with recombinant IFN-λs can protect from respiratory infections and stop the viral spread from upper to lower respiratory tract [33–36].

A genetic association was reported for *IFNL4*-rs12979860-T and rs368234815-dG alleles with impaired clearance of respiratory RNA viruses in nasal swabs of children from Rwanda [14]. In our study, we observed a borderline significant association (*p* = 0.045) with an earlier occurrence of respiratory infections in children with *IFNL4*-rs368234815-dG allele, but not reaching statistical significance. This could be because respiratory symptoms recorded during clinic visits in our study were defined very broadly, were very common (experienced by 97.7% of children during follow-up), and occurred very early in life, with the median timing of the first respiratory episode being at 16 weeks of age.

Although type III IFNs have been recognized for their antiviral role, their involvement in bacterial and fungal infections has also been reported [37]. Our findings on potential association with increased risk of malaria infection, which is caused by plasmodium, an intracellular parasite, supports the emerging role of type III IFNs beyond viral infections. Malaria plasmodium first invades hepatocytes and then propagates in the liver where type III IFNs can be expressed after induction by different stimuli. The results of earlier occurrence of clinical malaria episodes in children with the rs368234815-dG allele were recently corroborated by an independent study of children in Kenya [38].

While rs4803217 was previously suggested as a functional variant affecting the stability of *IFNL3* mRNA and possibly accounting for the association in the *IFNL3/IFNL4* region [13], this SNP appeared to be associated with all the outcomes in our study weaker than rs368234815, supporting *IFNL4*-rs368234815 as a primary associated and functional genetic variant in this region. The significant linear trends for associations between the inferred biological activity of IFN-λ4 (as Null-weak-strong IFN-λ4 protein) and infection episodes provide additional support to our hypothesis that IFN-λ4 activity increases the risk of acute clinical GII infections and malaria. We did not observe significant differences in the counts of infection episodes in children with various *IFNL4* genotype or IFN-λ4-P70S groups. Thus, the biological activity of IFN-λ4 is likely to be associated with increased overall risk and younger age at first clinical episodes but not with recurrence or intensity of infections.

The strength of our study includes its large sample size, longitudinal design, and detailed follow-up data collected over 30,626 clinic visits over almost 5 years that captured most, if not all, episodes of common infections in these children. The limitations of our study include using a syndromic approach of utilizing proxies for infections, such as diarrhea for gastrointestinal infections and cough/respiratory distress for respiratory infections, even though these infections could be caused by multiple factors [39]. Due to the regular medical assistance provided to the children at the time of routine and walk-in visits, the negative health impacts of these infections were likely to be diminished, potentially decreasing our statistical power to detect genetic associations with these infections. Our exploratory study tested associations between multiple infections and models, which might result in some of these results being false-positive due to multiple testing and unadjusted confounding factors.

Clearance of several RNA viruses associated with *IFNL4* genotype (HCV, respiratory and enteric viruses) suggests that response to other clinically relevant RNA viruses, including SARS-CoV-2 that causes COVID-19, might also be impaired in carriers of this genotype, and some support for this idea has recently been reported [40].

In conclusion, our study suggests that IFN-λ4 negatively affects the immune response to several infections in young children in Africa, in line with the overall unfavorable role of IFN-λ4 in various health conditions [12, 41]. Our results suggest new research directions to elucidate patterns of early childhood morbidity and mortality affected by IFN-λ4. Further studies in comparable cohorts are needed to confirm our findings and refine their etiology in different infectious, environmental and socio-economic settings.

## Supplementary information

Supplemental Material
