# Supplementary material for: IFN-λ4 is associated with increased risk and earlier occurrence of several common infections in African children
Source: Genes Immun. 2021 Apr 13;22(1):44–55. doi: 10.1038/s41435-021-00127-7 (PMC8042471; doi:10.1038/s41435-021-00127-7)
Supplement: Supplementary file 1 — Supplemental Material [file 41435_2021_127_MOESM1_ESM.pdf]

IFN-λ4 is associated with increased risk and earlier occurrence of several common infections in African children

Ludmila Prokunina-Olsson, Robert D. Morrison, Adeola Obajemu, Almahamoudou Mahamar, Sungduk Kim, Oumar Attaher, Oscar Florez-Vargas, Youssoufa Sidibe, Olusegun O. Onabajo, Amy A. Hutchinson, Michelle Manning, Jennifer Kwan, Nathan Brand, Alassane Dicko, Michal Fried, Paul S. Albert, Sam M. Mbulaiteye, Patrick E. Duffy

SUPPLEMENTARY MATERIALS

Table S1. Analysis of clinic visits reporting co-infections in 914 children from the Mali birth cohort study

Visit types	**Visits, N**	**Malaria & GII**	**Malaria & RI**	**GII & RI**
Routine visits with at least 1 infection	2377	19 (0.8%)	127 (5.3%)	58 (2.4%)
Walk-in visits with at least 1 infection	8303	136 (1.6%)	677 (8.2%)	444 (5.3%)

Table S2. Probability of co-infections reported at clinic visits in 914 children from the Mali birth cohort study, Log Odds

Routine visits with at least one infection

	Second infection		
First infection	Malaria	GII	RI
Malaria			
GII	-2.79		
RI	-3.35	-1.88	

Walk-in visits with at least one infection

	Second infection		
First infection	Malaria	GII	RI
Malaria			
GII	-2.03		
RI	-1.79	-2.6	

All logistic regression tests reported P-values < 2e-16 for Log Odds

Table S3. Distribution of *IFNL4*-rs368234815, *IFNL4*-rs117648444 and *IFNL3*-rs4803217 and *HBB* polymorphisms in populations of African ancestry from the 1000 Genomes Project and 914 children from the Mali birth cohort study

Gene-Variant	Alleles or Genotypes	ACB Caribbean N=96	ASW USA N=61	LWK Kenya N=99	ESN Nigeria N=99	GWD The Gambia N=113	MSL Sierra Leone N=85	YRI Nigeria N=108	All Mali N=914	Bamana Mali N=666	Peulh/ Fulani Mali N=84	Soninke Mali N=55	Malinke Mali N=46
***IFNL4*- rs11322783** corresponds to rs368234815 in 1000 Genomes	TT	0.26	0.3	0.44	0.28	0.22	0.29	0.27	0.29	0.28	0.36	0.27	0.29
	dG	0.75	0.7	0.56	0.72	0.78	0.71	0.73	0.71	0.72	0.64	0.73	0.71
	TT/TT	0.08	0.1	0.14	0.09	0.05	0.07	0.09	0.08	0.08	0.11	0.07	0.04
	TT/dG	0.34	0.41	0.61	0.38	0.34	0.44	0.35	0.41	0.39	0.50	0.39	0.50
	dG/dG	0.57	0.49	0.25	0.53	0.61	0.49	0.56	0.51	0.53	0.39	0.54	0.46
***IFNL3*- rs4803217**	G	0.29	0.34	0.47	0.28	0.3	0.38	0.33	0.33	0.33	0.42	0.27	0.29
	T	0.71	0.66	0.53	0.72	0.7	0.62	0.67	0.67	0.67	0.58	0.73	0.71
	G/G	0.1	0.15	0.18	0.07	0.09	0.15	0.12	0.11	0.11	0.15	0.07	0.04
	T/G	0.52	0.48	0.24	0.52	0.5	0.39	0.45	0.45	0.44	0.54	0.40	0.50
	T/T	0.38	0.37	0.58	0.41	0.41	0.46	0.43	0.44	0.45	0.31	0.53	0.46
***LD* between rs11322783 and rs4803217**	D’	0.97	1.00	0.85	0.92	1.00	1.00	1.00	0.98	0.98	1.00	0.95	1.00
	r^2	0.78	0.86	0.66	0.83	0.67	0.65	0.73	0.78	0.76	0.76	0.91	1.00
***IFNL4*- rs117648444**	A	0.09	0.06	0.07	0.05	0.11	0.04	0.10	0.10	0.10	0.13	0.09	0.12
	G	0.91	0.94	0.93	0.95	0.89	0.96	0.90	0.90	0.90	0.87	0.91	0.88
	A/A	0.01	0	0.01	0.03	0.01	0	0	0.01	0.01	0	0	0
	A/G	0.16	0.12	0.11	0.03	0.20	0.08	0.19	0.18	0.18	0.26	0.19	0.24
	G/G	0.83	0.88	0.88	0.94	0.79	0.92	0.81	0.81	0.81	0.74	0.81	0.76
***HBB*- rs334**	T	0.95	0.98	0.9	0.88	0.89	0.88	0.86	0.996	0.996	0.994	0.991	0.989
	A	0.05	0.02	0.1	0.12	0.12	0.12	0.14	0.004	0.004	0.006	0.009	0.011
	T/T	0.91	0.97	0.8	0.76	0.77	0.75	0.72	0.91	0.90	0.95	0.91	0.87
	A/T	0.09	0.03	0.2	0.24	0.23	0.25	0.28	0.09	0.096	0.048	0.091	0.13
	A/A	0	0	0	0	0	0	0	0.0011	0.002	0	0	0
***HBB*- rs33930165**	G	0.96	0.98	1.0	1.0	1.0	0.99	0.97	0.95	0.948	0.976	0.955	0.967
	A	0.04	0.02	0	0	0.004	0.006	0.030	0.050	0.052	0.024	0.045	0.033
	G/G	0.93	0.97	1.0	1.0	0.99	0.99	0.94	0.90	0.90	0.95	0.93	0.94
	G/A	0.07	0.03	0	0	0.009	0.010	0.060	0.096	0.104	0.048	0.055	0.065
	A/A	0	0	0	0	0	0	0	0.001	0	0	0.018	0

HBB status: WT – wild-type; SCT – sickle cell trait; SCD – sickle cell disease; 1000 Genomes Project populations of African ancestry (AFR) include Yoruba in Ibadan Nigeria (YRI), Luhya in Webuye, Kenya (LWK), Mandinka in The Gambia (MAG), Mende in Sierra Leone (MSL), Esan in Nigeria (ESN), Americans of African Ancestry in South-West USA (ASW), and African Caribbean in Barbados (ACB), based on information from https://www.internationalgenome.org/1000-genomes-browsers/. LD between markers was analyzed with LDLink (https://ldlink.nci.nih.gov/?tab=home). Mali dataset includes data for all 914 children and the four major tribes (based on the paternal tribe).

Table S4. Associations between *IFNL4*-rs368234815 and mutually adjusted risk of infections in 914 children from the Mali birth cohort study

Infection	Total<br>% of total	Ever/never*		Ever/never, adjusting for episodes of other infections					
		OR (95%CI)	P-val	OR (95%CI)	P-val	OR (95%CI)		OR (95%CI)	P-val
Malaria#	Never<br>23.9	Ref		Ref		Ref		Ref	
	Any<br>76.1	**1.32 (1.04-1.68)**	**0.022**	**1.31 (1.03-1.66)**	**0.030**	**1.3 (1.02-1.65)**	**0.034**	**1.31 (1.03-1.66)**	**0.031**
				Adjusted for GII		Adjusted for RI		Adjusted for GII and RI	
GII	Never<br>11.9	Ref		Ref		Ref		Ref	
	Ever<br>88.1	**1.5 (1.11-2.03)**	**0.0079**	**1.54 (1.14-2.09)**	**0.0050**	**1.49 1.1-2.01**	**0.0076**	**1.50 1.11-2.03**	**0.0069**
				Adjusted for malaria		Adjusted for RI		Adjusted for malaria and RI	
RI	Never<br>2.3	Ref		Ref		Ref		Ref	
	Ever<br>97.7	**1.2 0.63-2.29**	0.58	1.2 (0.63-2.29)	0.58	1.12 0.59-2.13	0.74	1.12 0.59-2.14	0.73
				Adjusted for malaria		Adjusted for GII		Adjusted for malaria and GII	

All the results are also adjusted for the paternal tribes.

Table S5. Time of follow-up (weeks) from birth in relation to *IFNL4*-rs368234815 in 914 children from the Mali birth cohort study

All children, weeks of follow-up Median (95% CI)	*IFNL4*-rs368234815 genotypes, weeks of follow-up, Median (95% CI)			P-value for non-parametric one-way ANOVA, Kruskal-Wallis test
	dG/dG	dG/TT	TT/TT	
140.0 (143.5-150.5)	142.5 (144.0-154.0)	140 (140.4-151.2)	143 (127.8-154.3)	0.537
Min-max time range, weeks 52-264	52-262	52-264	53-261	

Table S6. Relative rates of infection episodes per year in relation to *IFNL4*-rs368234815 genotypes during 1-5 years of follow-up in 914 children from the Mali birth cohort study

Infection	Max episode counts, N	Geometric mean*, N				Additive model#, Per-dG allele		Dominant model# dG/dG and dG/TT vs. TT/TT	
		All genotypes	dG/dG	dG/TT	TT/TT Ref	Relative rate	P-value	Relative rate	P-value
Malaria	22	2.82	2.84	2.86	2.45	1.08	0.14	1.17	0.21
SM	3	1.18	1.23	1.13	1.19	1.22	0.21	2.13	0.14
NSM	22	2.75	2.76	2.79	2.44	1.07	0.18	1.15	0.28
GII	14	2.49	2.51	2.54	2.20	1.05	0.23	1.22	**0.03**
RI	25	5.33	5.43	5.30	4.80	1.01	0.79	1.05	0.47

Malaria – includes both severe (SM) and non-severe malaria (NSM); GII – gastrointestinal infections; RI – respiratory infections. For malaria episodes – adjusted for *HBB* status. Geometric means were calculated excluding children who never experienced an infection episode (zero episodes). *Geometric mean is computed by exponentiating the average log-transformed number of episodes. The relative rate represents an increase in the number of episodes per year compared to the reference genotype group (TT/TT, IFN-λ4-Null), according to additive or dominant genetic models, and adjusting for the paternal tribes.

Table S7. Associations between *IFNL3*-rs4803217 and infections in 914 children from the Mali birth cohort study

Infections			rs4803217 genotypes			rs4803217 alleles		Ever/never*		Ever/never$	
	Total	Total	G/G	T/G	T/T	OR	OR				
	% of total	n, %	n , %	n , %	n , %	n, %	n, %	OR	P-val	OR	P-val
		914	101	407	406	609	1219				
			11.5	44.5	44.4	33.3	66.7				
Malaria#	Never	218	29	102	87	160	276	Ref		Ref	
	23.9		13.3	46.8	39.9	36.7	63.3				
	Any	696	72	305	319	449	943	1.2	0.122	1.19	0.15
	76.1		10.3	43.8	45.8	32.3	67.7	(0.95-1.52)		(0.94-1.50)	
	SM	102	4	48	50	56	148	**1.56**	**0.032**	**1.52**	**0.044**
	11.2		3.9	47.1	49	27.5	72.5	**(1.04-2.33)**		**(1.01-2.27)**	
	NSM	594	68	257	269	393	795	1.16 (0.92-1.47)	0.22	1.15 (0.91-1.46)	0.25
	65		11.4	43.3	45.3	33.1	66.9				
GII	Never	109	16	54	39	86	132	Ref			
	11.9		14.7	49.5	35.8	39.4	60.6				
	Ever	805	85	353	367	523	1087	1.34 (0.99-1.80)	0.055	**1.35 (1.00-1.82)**	**0.047**
	88.1		10.6	43.9	45.6	32.5	67.5				
RI	Never	21	4	9	8	17	25	Ref			
	2.3		19	42.9	38.1	40.5	59.5				
	Ever	893	97	398	398	592	1194	1.24	0.51	1.24	0.51
	97.7		10.9	44.6	44.6	33.1	66.9	(0.66-2.33)		(0.66-2.33)	

***** Logistic regression analysis based on additive genetic models and adjusting for the duration of follow-up (weeks); #results for malaria are also adjusted for *HBB* status; $ - additionally adjusting for the paternal tribes; GII – gastrointestinal infections; RI – respiratory infections; any malaria – positivity by blood smear test with/without additional clinical symptoms; SM – severe malaria; NSM – non-severe malaria; in bold – significant associations.

Table S8. Relative rates of infection episodes per year in relation to *IFNL4*-P70S groups during 1-5 years of follow-up in 914 children from the Mali birth cohort study

Infection	Max episode counts, N	Geometric mean*, N of episodes in IFN-λ4-P70S groups					Additive model#, per group	
		All genotypes	Strong P70 2 copies	Strong P70 1 copy	Weak, S70	Null	Relative rate	P-value
Malaria	22	2.82	2.83	2.80	3.28	2.45	1.05	0.22
SM	3	1.18	1.21	1.13	1.44	1.19	1.13	0.33
NSM	22	2.75	2.75	2.73	3.22	2.44	1.05	0.25
GII	14	2.49	2.46	2.60	2.33	2.20	1.05	0.07
RI	25	5.33	5.38	5.42	5.08	4.80	1.01	0.54

Malaria – includes both severe (SM) and non-severe malaria (NSM); GII – gastrointestinal infections; RI – respiratory infections. For malaria episodes – adjusted for *HBB* status. Geometric means were calculated excluding children who never experienced an infection episode (zero episodes). *Geometric mean is computed by exponentiating the average log-transformed number of episodes. We fitted the Poisson model with overdispersion (Quasi-likelihood) with offset = log(LastVisitAge). The relative rate represents an increase in the number of episodes per year per group compared to the reference genotype group (IFN-λ4-Null), according to additive genetic model, and adjusting for the paternal tribes. IFN-λ4-P70S genotype groups are described in **Table 5**.

SUPPLEMENTARY FIGURES

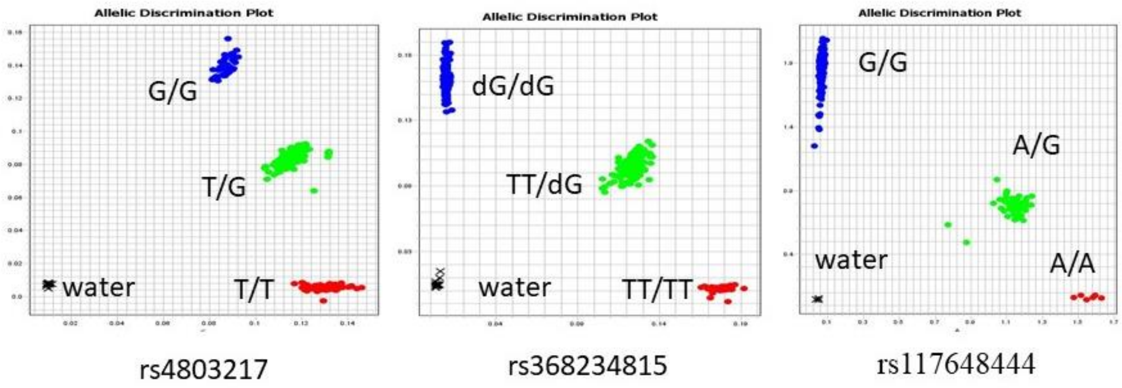

Figure S1. Representative allelic discrimination plots for genotyping *IFNL4*-rs368234815, *IFNL4*-rs117648444 and *IFNL3*-rs4803217 in the Mali birth cohort study samples by custom TaqMan genotyping assays.

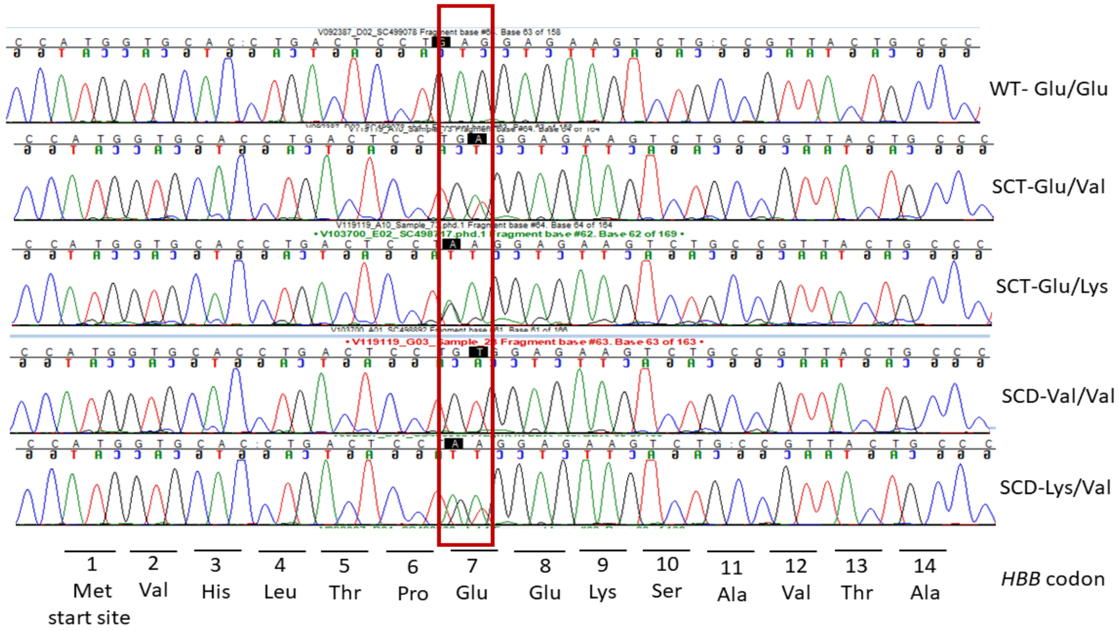

Figure S2. Representative Sanger sequencing chromatograms for genotyping of the *HBB* polymorphisms rs33930165-A/G (Glu7Lys) and rs334-A/T (Glu7Val) in the Mali birth cohort study samples.

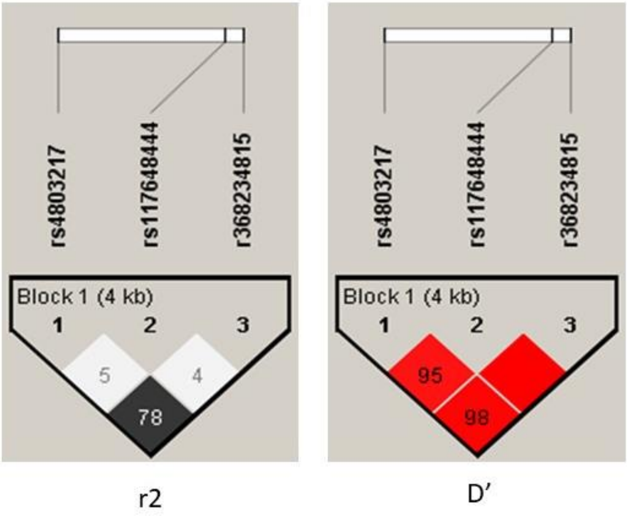

**Haplotypes of *IFNL3*-rs4803217, *IFNL4*-rs117648444 and *IFNL4*-rs368234815 in 914 children from the Mali birth cohort study**					
Haplotype	Frequency, %	*IFNL3* 3'UTR rs4803217	*IFNL4*-rs117648444 P70S	*IFNL4* frame-shift rs368234815	IFN-λ4 protein
1	56.6	T	G	dG	Produced, strong IFN-λ4-P70
2	28.5	G	G	TT	Not produced IFN-λ4-Null
3	9.9	T	A	dG	Produced, weak IFN-λ4-S70
4	4.7	G	G	dG	Produced, strong IFN-λ4-P70

Figure S3. HaploView linkage disequilibrium (LD) plots and haplotypes of *IFNL3*-rs4803217, *IFNL4*-rs117648444 and *IFNL4*-rs368234815 and in 914 children from the Mali birth cohort study. The plots show LD metrics - r^2 and D'.

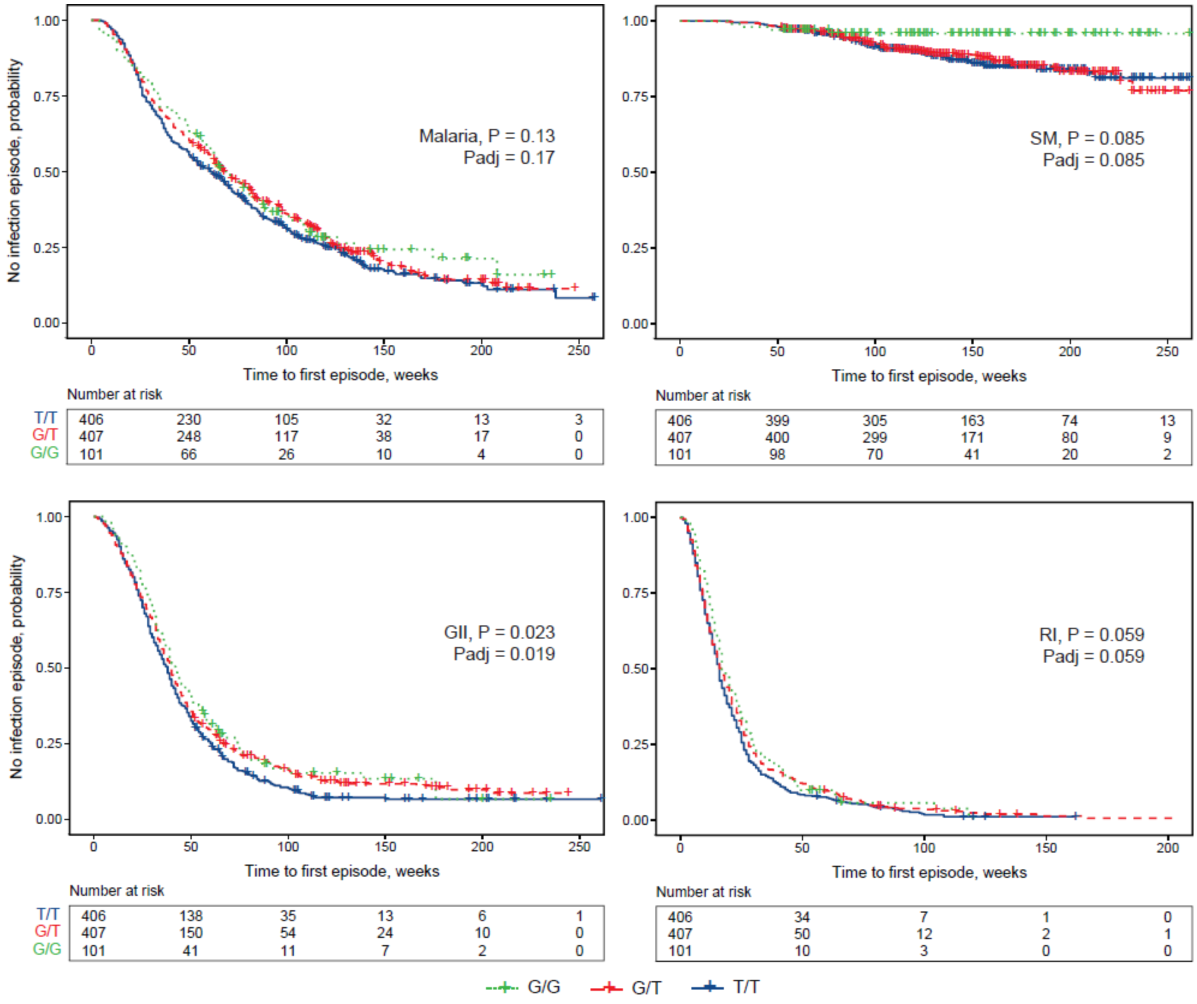

Figure S4. Time-to-first episode of infections in relation to *IFNL3*-rs4803217 SNP in 914 children from the Mali birth cohort study. Malaria – positivity by blood smear test with/without additional clinical symptoms, includes both severe (SM) and non-severe malaria; GII – gastrointestinal infections; RI – respiratory infections. The plots are for Kaplan-Meyer analysis, P-values are for Cox proportional hazards regression models with per-allele linear trends, for malaria – adjusting for *HBB* status. Padj - additionally adjusting for the paternal tribes.
